# Apical Gap, Adaptation, and Trueness of Direct and Indirect Customized Post‐and‐Core Restorations Fabricated by the Casting, Milling, and Selective Laser Melting Techniques

**DOI:** 10.1155/ijod/4618908

**Published:** 2026-07-17

**Authors:** Hadi Ranjzad, Jalal Toumaj, Farzaneh Ostovarrad, Mehran Falahchai, Mahyar Eftekhar

**Affiliations:** ^1^ Department of Prosthodontics, Dental Sciences Research Center, School of Dentistry, Guilan University of Medical Sciences, Rasht, Iran, gums.ac.ir; ^2^ Department of Prosthodontics, School of Dentistry, Golestan University of Medical Sciences, Gorgan, Iran, goums.ac.ir; ^3^ Department of Oral and Maxillofacial Radiology, Dental Sciences Research Center, Guilan University of Medical Sciences, Rasht, Iran, gums.ac.ir

**Keywords:** computer-aided design, computer-aided manufacturing, dental casting technique, dimensional measurement accuracy, lasers, post-and-core, technique

## Abstract

**Background:**

Customized post‐and‐core restorations can be fabricated using direct or indirect workflows, combined with conventional casting or digital techniques, including milling and selective laser melting (SLM). However, evidence on how these approaches influence apical gap, adaptation, and trueness remains limited. This study aimed to evaluate these outcomes in direct and indirect post‐and‐core restorations fabricated by casting, milling, and SLM.

**Methods:**

Eighty‐four mandibular molar post‐and‐core restorations were fabricated in six groups (*n* = 14) combining two methods (direct and indirect) with three techniques (casting, milling, and SLM). The apical gap was measured on parallel digital radiographs; adaptation was assessed by quantifying the mass of light‐body silicone captured between the post and canal walls; trueness was calculated as root mean square (RMS) deviation in Geomagic. Data were analyzed using mixed‐effects models with Bonferroni‐adjusted pairwise and paired *t*‐tests (*α* = 0.05).

**Results:**

For apical gap, fabrication technique had no significant effect within either workflow (direct: *p* = 0.639; indirect: *p* = 0.138); whereas, direct casting demonstrated a significantly smaller apical gap than indirect casting (*p* = 0.009). For adaptation, direct casting showed superior performance compared with direct milling (*p* = 0.050); in contrast, indirect SLM demonstrated better results than indirect casting (*p* = 0.036); direct casting also yielded better adaptation than indirect casting (*p*  < 0.001). For trueness, direct milling was less true than direct casting or SLM (*p*  < 0.001); indirect casting was less true than indirect milling or SLM (*p*  = 0.011). Across all groups, RMS values were evaluated against the conservative (0.05 mm) and permissive (0.10 mm) thresholds. All specimens (84/84, 100%) exceeded 0.05 mm, and 72 of 84 specimens (85.7%) exceeded 0.10 mm, with group‐level proportions above 0.10 mm, ranging from 71.4% (casting) to 100% (milling).

**Conclusions:**

Both fabrication technique (casting, milling, and SLM) and method (direct and indirect) affected apical gap, adaptation, and trueness. The most favorable combinations were direct casting and indirect SLM.

## 1. Background

Restoration of endodontically treated teeth with severe coronal destruction often requires post‐and‐core restorations [1–5]. Intracanal posts can be fabricated from fiber or metal, with comparable clinical survival and success rates [1, 6–8]. However, in flared or irregular canals, cast metal post‐and‐core restorations are preferred due to their superior adaptation to canal morphology and greater fracture resistance and tolerance to torsional forces [9, 10]; although concerns remain regarding corrosion and the risk of nonrestorable root fractures [1, 11, 12].

Cast metal posts can be fabricated by either direct (intraoral resin pattern) or indirect (laboratory‐made pattern from elastomeric impressions) methods [13, 14]. Evidence regarding the adaptation and accuracy of these methods is conflicting, with some studies reporting similar performance [15] and others showing better adaptation with the direct method [16].

Recent advances in computer‐aided design and computer‐aided manufacturing (CAD/CAM) provide alternative techniques for post‐and‐core fabrication. Subtractive manufacturing, such as milling, removes material from a solid block to shape the restoration. Additive manufacturing builds structures layer by layer and includes photopolymerization‐based methods such as stereolithography and digital light processing, as well as powder bed fusion methods, including selective laser sintering and selective laser melting (SLM) [17–25]. Additive techniques produce less material waste, and allow fabrication of geometrically complex structures that may be inaccessible to milling burs [19–22, 26]. Among these, SLM has been increasingly applied in dentistry for the fabrication of a wide range of restorations and prosthetic components, including crowns, fixed dental prostheses, removable partial denture frameworks, and post‐and‐core systems [27, 28]. SLM enables precise fabrication of intricate geometries and may improve adaptation and trueness compared with conventional casting or milling, although evidence remains inconsistent [27, 29, 30].

Two digital workflows have been described for impression acquisition in post‐and‐core fabrication: a fully digital workflow based on scan‐post acquisition and virtual model generation and a semi‐digital workflow involving conventional impression or pattern fabrication followed by laboratory scanning [31, 32]. In the fully digital approach, intraoral or laboratory scanning of the post space is used to directly capture the internal geometry and generate a virtual model, thereby eliminating conventional impression procedures [33]. Whereas this workflow streamlines the clinical and laboratory processes, its accuracy may be affected by limitations in capturing complex intracanal geometries and potential errors in scan‐post registration and alignment [34, 35]. In contrast, the semi‐digital workflow, which uses resin patterns or silicone impressions followed by laboratory scanning, allows more reliable reproduction of the intracanal morphology [31, 32], although it involves additional procedural steps. Selection between these approaches reflects a balance between efficiency and geometric accuracy, particularly in challenging clinical scenarios [33, 36, 37].

Key parameters for evaluating post‐and‐core restorations include apical gap, adaptation, and trueness, which reflect the seating accuracy, cement thickness, and overall fit [29, 30, 38–40]. In the present study, the apical gap was assessed using standardized periapical radiographs due to their wide availability and reproducibility in apical assessment under clinical‐like conditions, although they are limited to two‐dimensional evaluation [31, 41]. Adaptation was evaluated by measuring the weight of light‐body silicone material trapped between the post and canal walls, providing an indirect but widely used method for assessing the internal fit [39]. Trueness was determined through digital scanning and three‐dimensional (3D) superimposition analysis using Geomagic software, with root mean square (RMS) deviation calculated and color‐coded deviation maps generated for visualization, as described in detail in the Section 2 [42]. Alternative methods reported in the literature include micro‐computed tomography for volumetric gap analysis and silicone replica techniques combined with 3D superimposition, both of which provide more comprehensive spatial evaluation of internal and marginal discrepancies [43].

Considering the variability in the existing evidence regarding SLM and other fabrication methods, the aim of this in vitro study was to evaluate the apical gap, adaptation, and trueness of direct and indirect customized post‐and‐core restorations fabricated using casting, milling, and SLM techniques. The null hypothesis was that there would be no significant differences among the fabrication methods and techniques in any of the measured parameters.

## 2. Materials and Methods

Sample size was estimated using 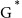
Power software (Version 3.1.9.2; Heinrich Heine University, Düsseldorf, Germany) based on an *F*‐test for repeated‐measures analysis of variance. An effect size of *f* = 0.36 was assumed, with a significance level (*α*) of 0.05 and a statistical power (1–*β*) of 0.80 using a two‐tailed test. A correlation of 0.5 among the repeated measurements was assumed. The required sample size was calculated as 14 specimens per group [14]. Thus, a total of 14 mandibular first molars recently extracted due to periodontal reasons were collected for this in vitro study. The study was approved by the Research Ethics Committee of Guilan University of Medical Sciences (IR.GUMS.REC.1402.022), and written informed consent was obtained from all patients.

The teeth were selected based on clinical and radiographic examinations using the following inclusion criteria: absence of caries and the presence of distinct mesial and distal roots. The exclusion criteria were cracks or restorations and structural defects such as root resorption, calcification, obstruction, incomplete apex, and curvature of the distal root [44]. The teeth had almost similar dimensions, with a buccolingual width of 9.40 ± 0.60 mm, a mesiodistal width of 11.20 ± 0.80 mm, and a root length of 14.80 ± 0.80 mm, as measured by a digital caliper (Mitutoyo, Tokyo, Japan). External debris, calculus, and soft tissue residues were removed with an ultrasonic scaler (UltraMint Pro; Eighteeth Medical Technology Co., Ltd., Changzhou, China), followed by cleaning with a prophylaxis paste. The teeth were stored in 0.10% thymol solution for 2 weeks, then transferred to distilled water, and incubated at 4°C until the experiment.

Each tooth was sectioned 2 mm coronal to the cementoenamel junction, parallel to the occlusal plane, using a diamond rotary instrument under water irrigation. The specimens were then embedded in auto‐polymerizing acrylic resin blocks (Palapress; Heraeus Kulzer, Hanau, Germany; 20 × 20 mm), with the cementoenamel junction positioned 2 mm above the surface of the resin block.

Following access cavity preparation, the working length was determined by using a #15 stainless‐steel K‐file (Mani Corp., Tochigi, Japan) and confirmed radiographically. The root canals were cleaned, shaped, and instrumented with a nickel–titanium rotary system (ProTaper Next; Dentsply Maillefer, Ballaigues, Switzerland) using the crown‐down technique, with saline irrigation throughout the procedure [45]. After preparation, the canals were dried with air spray and paper points (Autofit; Kerr Corporation, Orange, CA, USA) and obturated with gutta‐percha (Gutta Percha Points; Meta Biomed Co.) and AH26 sealer (Dentsply DeTrey GmbH, Konstanz, Germany) using the lateral compaction technique [45]. Using a standardized technique, a single qualified operator performed each root canal therapy procedure.

A No. 2 peeso reamer (Ultradent Products, Inc., South Jordan, UT, USA) was used to prepare post spaces, and its working length was adjusted to leave 5 mm of apical gutta‐percha in each specimen [46]. All preparations were performed by one single operator following a standardized protocol to ensure the uniformity of shape and taper.

For each tooth, a post was made once using the direct method and once using the indirect method.

To minimize potential bias related to repeated procedures on the same canal, the order of the workflows was randomized and counterbalanced across specimens so that neither method was consistently performed first. In the direct method, the prefabricated plastic post (PinJet; Angelus, Londrina, Brazil) was relined with a thick mixture of auto‐polymerizing acrylic resin (Pattern Resin LS; GC Corporation, Tokyo, Japan) and inserted into the post space; a core part with a 4 mm height and an anatomical occlusal table was also formed. Using the same resin material and regular operating techniques, a single qualified operator fabricated the resin patterns. Prior to fabricating the study samples, the operator ran pilot patterns to standardize the shape and the seating of the patterns.

Next, the resin patterns were digitized by a single trained operator using a laboratory scanner (AutoScan DS‐EX Pro; Shining 3D Technology Co., Ltd., Hangzhou, China) to obtain a standard‐tessellation‐language (STL) file. Scanner calibration was performed before each session. Subsequently, the post‐and‐core models were designed using Exocad software (Exocad GmbH, Darmstadt, Germany; Version 2.4) with a cement space of 20 μm [31]. Design parameters (resolution/mesh density) followed the manufacturer’s recommendations and were kept constant for all samples. The post‐and‐core restorations were milled from a cobalt–chromium (Co–Cr) alloy (Ceramill Sintron R71 CoCrMo disc; Amann Girrbach AG, Vorarlberg, Austria) using a 5‐axis dental milling machine (Ceramill Motion 2; Amann Girrbach AG, Vorarlberg, Austria) by a trained dental technician. For tool wear monitoring, all carbide burs were length‐calibrated by the Ceramill Motion 2’s built‐in probe before each build. Worn burs were discarded, and a new, factory‐sharpened bur was installed before milling proceeded. After the milling process, each post‐and‐core underwent sintering at 1500°C for a duration of 5 h and 10 min in a sintering furnace (Ceramill Argotherm 2; Amann Girrbach AG, Vorarlberg, Austria), as per the manufacturer’s instructions. Additionally, the 3D‐printed post‐and‐core restorations were produced using a SLM machine (Riton D‐100; Guangzhou Riton 3D Technology Co., Ltd., Guangzhou, China) by a trained dental technician. All specimens were fabricated in a vertical build orientation, with the longitudinal axis perpendicular to the build platform, to ensure consistency in dimensional accuracy. The key processing parameters included a power of 220 W, a 30‐µm layer thickness, a linear scan speed of 900 mm/s, and a 100‐µm laser‐spot diameter. Gas‐atomized Co–Cr–Mo powder feedstock (61.50 wt % Co, 27.75 wt % Cr, 9 wt % W, traces of Fe and Si; Riton RXT‐01, China) was first sieved to 15–53 µm; its laser‐diffraction profile was *D*_10_ ≥ 12 µm and *D*_90_ ≤ 65 µm, with flowability ≤ 40 s ∙ 50 g, apparent density ≥ 4.0 g cm^−3^ and tap density ≥ 4.5 g cm^−3^. Only virgin powder was used for all builds to eliminate the variability associated with powder reuse. Before fabrication, the build chamber was repeatedly purged with high‐purity argon until the residual oxygen concentration fell below 0.05%, and this level was maintained throughout the printing process. Support structures were automatically generated in Voxel Dance Additive v4.1 using the default dental template, with a medium support density to ensure adequate thermal stability during fabrication. After printing, the parts were removed from the build plate using a low‐speed diamond disc, and supports were trimmed with carbide burs. A stress‐relief heat treatment (1000°C for 60 min under an argon atmosphere) was then applied, followed by furnace cooling.

To produce cast posts, the post‐and‐core resin patterns were invested with a phosphate‐bonded investment material (Z4 Universal Rapid Cast Investment; Neirynck & Vogt nv, Belgium). For the investment, each 100 g of powder was mixed with 24.00 mL of water, and the expansion of the investment was controlled according to the manufacturer’s recommended liquid/powder ratio. The investment material was initially mixed manually with a hand‐held spatula for 30 s, followed by vacuum mixing at 350 rpm for 60 s to minimize porosity. The casting ring was then filled and allowed to set for 30 min at room temperature.

Each invested post‐and‐core resin pattern was placed in a burnout furnace (Programix 50 Burnout Furnace; Ugin Dentaire, Seyssinet‐Pariset, France) and subjected to a programmed burnout cycle: heating from room temperature to 900°C at a rate of ~10°C/min, followed by a holding time of 60 min to ensure complete elimination of the acrylic resin pattern. Casting was performed using a centrifugal casting system (Ducatron S3; Ugin Dentaire, Seyssinet‐Pariset, France) in accordance with the manufacturer’s instructions. The Co–Cr alloy (62.50%wt Co; 24.60%wt Cr; 8.50%wt W; 2.90%wt Mo; and trace amounts of Fe and Si; Realloy‐C, Realloy, Germany) was melted to a casting temperature of 1450°C and cast into the mold. After casting, the mold was allowed to bench cool to room temperature prior to divesting. All casting procedures were performed by a trained dental technician. It should be noted that the alloy compositions used for SLM fabrication and conventional casting were not chemically identical. Therefore, potential differences in material properties could not be completely controlled across fabrication techniques.

**Figure 1:**
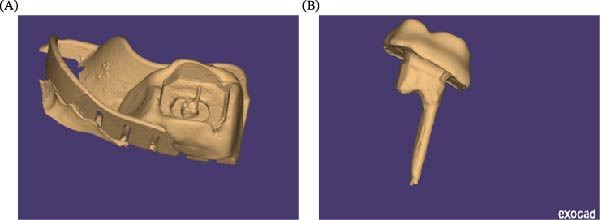
STL data of the indirect method for fabricating post‐and‐core restorations. (A) Silicone impression of the prepared canal. (B) Designed post‐and‐core model. STL, standard‐tessellation‐language.

In the indirect method, a one‐stage two‐phase impression was made using a light‐body polyvinyl siloxane impression material (Vario Light Body; Betasil; Muller‐Omicron GmbH & Co.KG, Germany) and a heavy‐body material (Vario; Betasil; Muller‐Omicron GmbH & Co.KG, Germany). The heavy‐body material was mixed and inserted into the tray. Simultaneously, the light‐body material was mixed and injected into the post space using a lentulo paste carrier (Dentsply Sirona, Ballaigues, Switzerland/York, PA, USA), followed by the placement of a prefabricated plastic post (Angelus Pinjet) in the canal. Finally, the impression tray was placed over the tooth. Impressions were made by a trained operator. Then, the impression was digitized using a laboratory scanner (Shining 3D, AutoScan DS‐EX Pro) to obtain an STL file. Subsequently, the post‐and‐core models were designed using Exocad software with a 20‐μm cement space (Figure 1).

The Co–Cr post‐and‐core restorations were manufactured using the milling technique and also the SLM technique, following the same processes as mentioned for the direct method. Moreover, the impressions were poured using type IV dental stone (GC base stone; GC, Japan) to fabricate dental casts. Subsequently, after lubricating the casts with a thin layer of petroleum jelly (Vaseline; Unilever, London, UK), the post‐and‐core resin patterns were formed with an auto‐polymerizing acrylic resin (Pattern Resin LS; GC) by a trained operator. Before producing the study specimens, the operator conducted calibration trials to ensure a consistent pattern geometry and dimensional accuracy. These patterns were cast using the Co–Cr alloy with the same process as mentioned for the direct method.

For standardization and environmental control, the entire manufacturing workflow, encompassing pattern fabrication, laboratory scanning, milling, SLM printing, and postprocessing, was conducted in one single climate‐controlled laboratory. The laboratory maintained a temperature of 22 ± 1°C and a relative humidity of 45% ± 5%. The temperature and humidity were recorded twice daily using a digital thermo‐hygrometer. Postprocessing was identical for all Co–Cr posts. Stress‐relief heat treatment was performed by heating the posts at 1000°C for 60 min under an argon atmosphere. Subsequently, airborne‐particle abrasion was performed using 250 µm Al_2_O_3_ particles (Basic classic; Renfert GmbH, Hilzingen, Germany) at 0.3 MPa for 10 s. Finally, ultrasonic cleaning was conducted to ensure the posts were thoroughly cleaned. A trained operator adjusted all the fabricated post‐and‐core restorations using a Fit Checker (Fit Checker II, GC America) to ensure a passive fit during insertion into the canals.

**Figure 2:**
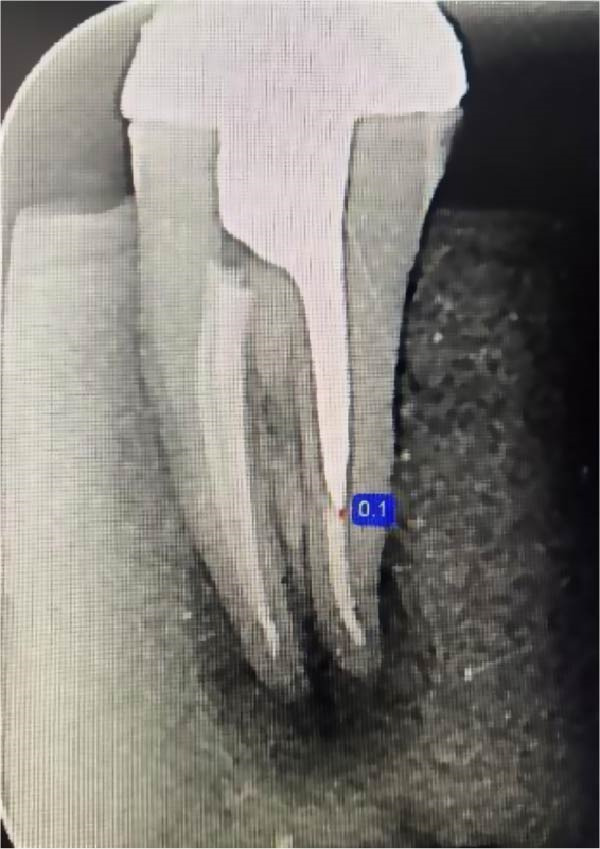
Apical gap measurement using Planmeca Romexis viewer.

The apical gap was defined as the distance from the tip of the post to the remaining gutta‐percha [47]. To measure the apical gap, standardized periapical radiographs were obtained using a photostimulable phosphor plate sensor with a parallel technique. The X‐ray unit (Planmeca ProX; Planmeca, Helsinki, Finland) was set at 60 kVp, 1 mA, and 0.08 s exposure time, with a fixed source‐to‐object distance of 25 cm and a standardized beam geometry for all samples. Each radiograph was calibrated using the known length of the post as a reference scale in the Planmeca Romexis software (Planmeca, Helsinki, Finland) (Figure 2). The apical gap was measured by a blinded, experienced oral and maxillofacial radiologist. Each measurement was repeated twice with a 2‐week interval, and the mean value was recorded as the final measurement. Intraexaminer reliability was assessed using the intraclass correlation coefficient (ICC = 0.90), indicating excellent agreement.

**Figure 3:**
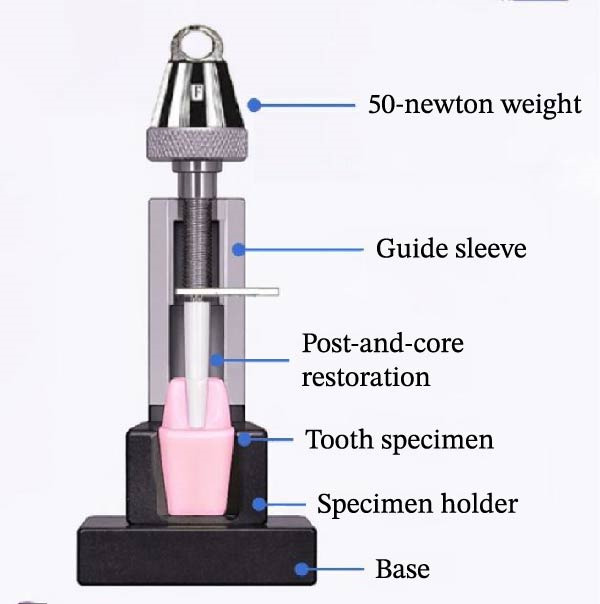
Schematic picture of the custom‐made positioning jig.

To evaluate post adaptation, the weight of light‐body silicone impression material (Betasil) trapped between the post and canal wall was measured [48]. The silicone material has a manufacturer‐reported density close to 1 g/cm^3^, allowing its weight to be considered an indirect estimation of its volume. After canal lubrication, the light‐body material was injected into the post space. To ensure consistent orientation and seating depth, the post‐and‐core restoration was positioned using a custom jig (Figure 3), which guided the post along its longitudinal axis and minimized lateral deviation. A 50 N force was applied to the platform of the post‐and‐core via an orangewood stick and maintained for approximately 5 min until polymerization was complete. Following polymerization, the assembly was examined at a magnification of x 10; any sample exhibiting folds, flash, or voids was thrown away, and the process was repeated.

Once the impression material was polymerized (~5 min), excess material beyond the core margins was trimmed with a scalpel (Techno Cut Scalpel; HMD Healthcare Ltd., India). The post was then removed carefully from the canal, and the weight of silicone material was measured by using a digital analytical balance (GH Series; A&D Company Ltd., Tokyo, Japan) with 0.0001 g accuracy. All measurements were made by one single operator blinded to group allocation.

Trueness was defined as the level of agreement between the reference and the scanned post.

For measuring the trueness, the following technique was used with the help of Geomagic Quality software version 2020 (3D Systems, Rock Hill, SC, USA).

To create STL files, the resin patterns—serving as the reference geometry for each specimen—were digitized with an accuracy of 8–10 µm using a laboratory scanner (AutoScan DS‐EX Pro; Shining 3D, Hangzhou, China). In the direct method, these resin patterns were used both as the reference for digital scanning and as the templates for conventional casting procedures. The scans were obtained using the maximum detail setting and in accordance with the manufacturer’s recommended workflow, including calibration prior to each scanning session. All scans were performed by one single trained operator experienced in laboratory scanning procedures. To ensure repeatability, calibration scans of the manufacturer’s reference object were conducted before data acquisition. The resulting STL files of the resin patterns (reference) were then compared with the STL files of the corresponding fabricated metal post‐and‐core restorations using Geomagic Quality software version 2020 (3D Systems, Rock Hill, SC, USA).

**Figure 4:**
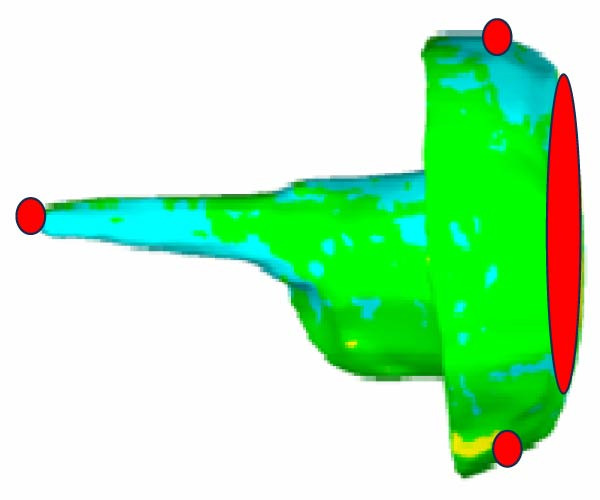
Landmarks used for initial global alignment: post’s apical tip, the flattened occlusal surface of the core, and two peripheral reference points on the lateral surfaces.

There were two steps involved in the alignment. Initially, a global (coarse) alignment was performed using predefined anatomical and geometric landmarks, including the post’s apical tip, a flat surface on the coronal portion of the core (defined as the intentionally flattened occlusal surface created during pattern fabrication), and two peripheral reference points located on the lateral surfaces of the core to provide spatial orientation (Figure 4).

Following landmark‐based alignment, global best‐fit and iterative closest point refinement were applied. The whole‐surface best‐fit algorithm was then used to align the entire object through iterative closest‐point refinement. Sectioning‐based masking was applied to exclude regions outside the post portion from alignment in order to reduce the influence of irrelevant areas, such as excessive core anatomy. Following alignment, point‐to‐point deviations were calculated, and the RMS deviation was computed using the following formula to represent trueness:

RMS=∑i=1nX1,i−X2,i2n.

In this formula, “*n*” represents the number of corresponding points, “X1.i” denotes the reference points in the STL files of the resin patterns, and “X2.i” refers to the corresponding points in the STL files of the fabricated post‐and‐core restorations.

Color‐coded deviation maps were generated using a −0.50 to + 0.50 mm scale to visualize the overall distribution of surface deviations between the reference and fabricated models. These maps were used for qualitative visualization only. A quantitative assessment of trueness was performed using RMS values calculated from the entire surface deviation data. The alignment and RMS workflow adhered to accepted metrology practices and commonly used reference thresholds for clinical interpretation (0.05 and 0.10 mm) that are frequently reported in the literature. Both values were considered when interpreting the results as commonly used reference thresholds to aid clinical interpretation under both conservative and permissive criteria. The 0.05‐ and 0.10‐mm thresholds were applied solely for the interpretation of these RMS values and not for the color‐map display [49, 50].

In terms of the blinding process, each specimen was given a random code prior to any measurements by an impartial technician who was not involved in the data collection. The key linking codes to experimental groups was stored by the senior investigator and released only after all statistical analyses had been completed. Therefore, during data collection, the engineer performing STL superimposition for trueness, the operator recording silicone weight for adaptation, and the oral‐maxillofacial radiologist measuring the apical gap all worked solely with anonymized specimens or digital files and were not aware of the fabrication technique or direct/indirect allocation.

Regarding the reliability procedure, all measurements were performed independently by two blinded examiners. After completing their separate readings, the results were compared; if any discrepancy was detected, the specimen or file was re‐examined, and a consensus value was recorded. In addition, inter‐ and intraobserver reliability were assessed using the weighted kappa statistic.

The normality of data distribution for adaptation, apical gap, and trueness (RMS) was assessed using the Kolmogorov–Smirnov test. Statistical comparisons between workflows (direct and indirect) were performed using the paired *t*‐test. Comparisons among the fabrication techniques (casting, SLM, and milling) were conducted using mixed‐effects models with an unstructured covariance matrix. Pairwise comparisons were adjusted for multiple testing using the Bonferroni correction. Effect sizes were reported as partial *η* ^2^ for mixed‐effects models and Cohen’s d for paired *t*‐tests. Statistical analyses were conducted using IBM SPSS Statistics Version 28.0 (IBM Corp., Armonk, NY, USA), and the level of significance was set at *α* = 0.05.

## 3. Results

The mean and standard deviation of apical gap, adaptation, and trueness (RMS) for each group are presented in Table 1. For apical gap, the mixed‐effects analysis showed no significant difference among the fabrication techniques in the direct groups (F[2,39] = 0.453, *p* = 0.639, partial *η* ^2^ = 0.02). Similarly, no significant difference was observed among the techniques in the indirect groups (F[2,39] = 2.082, *p* = 0.138, partial *η* ^2^ = 0.10). In comparison of the apical gap between the direct and indirect methods across different fabrication techniques, paired *t*‐test revealed a significant difference only in the casting group, where the indirect method demonstrated a higher apical gap than the direct method (mean difference = −0.14 mm; 95% CI: −0.23 to −0.04; *p* = 0.009; Cohen’s *d* = 0.82).

**Table 1:** Comparison of apical gap (mm), adaptation (mg), and trueness (RMS, mm) in different fabrication techniques (casting/milling/SLM) across different fabrication methods (direct/indirect).

Outcome measure	Workflow		Fabrication technique						*p*‐Value (partial *η* ^2^)
			Casting Mean ± SD		SLM Mean ± SD		Milling Mean ± SD		
Apical gap (mm)	Direct		0.33 ± 0.23		0.31 ± 0.20		0.39 ± 0.20		0.639 (0.02)
	Indirect		0.46 ± 0.24		0.31 ± 0.15		0.34 ± 0.22		0.138 (0.10)
MD (95% CI)	—		−0.14 (−0.23, −0.04)		0 (−0.06, 0.06)		0.04 (−0.02, 0.11)		—
*p*‐Value (Cohen’s *d*)	—		0.009 (0.82)		>0.999 (0)		0.189 (0.37)		—
Adaptation (mg)	Direct		45.30 ± 12.6		52.55 ± 12.14		56.48 ± 10.60		0.050 (0.14)
	Indirect		67.05 ± 12.72^a^		52.56 ± 14.84^b^		62.91 ± 15.84^ab^		0.034 (0.16)
MD (95% CI)	—		−21.75 (−27.8, −15.70)		−0.007 (−4.78, 4.77)		−6.43 (−14.83, 1.97)		—
*p*‐Value (Cohen’s *d*)	—		<0.001 (2.08)		0.997 (0.001)		0.122 (0.44)		—
Trueness (RMS; mm)	Direct		0.09 ± 0.04^a^		0.10 ± 0.03^a^		0.14 ± 0.02^b^		<0.001 (0.38)
	Indirect		0.13 ± 0.03^a^		0.08 ± 0.02^b^		0.10 ± 0.06^ab^		0.013 (0.20)
MD (95% CI)	—		−0.04 (−0.06, −0.01)		0.02 (−0.002, 0.03)		0.04 (0.002, 0.07)		—
*p*‐Value (Cohen’s *d*)	—		0.007 (0.85)		0.083 (0.50)		0.038 (0.62)		—
*p*‐Value (Cohen’s *d*)	—		0.007 (0.85)		0.083 (0.50)		0.038 (0.62)		—

*Note: N* = 14 per group. Statistical comparisons between workflows (direct and indirect) were conducted using paired *t*‐tests. Comparisons among fabrication techniques (casting, SLM, milling) were performed using mixed‐effects models with an unstructured covariance matrix. Pairwise comparisons between techniques were corrected for multiple comparisons using the Bonferroni adjustment. Effect sizes are reported as partial *η* ^2^ for mixed‐effects models and Cohen’s *d* for paired *t*‐tests. Different superscript letters (e.g., a, b) indicate statistically significant differences between fabrication techniques (*p*  < 0.05, Bonferroni‐adjusted).

Abbreviations: CI, confidence interval; MD, mean difference; RMS, root mean square.

For adaptation, the mixed‐effects model showed a borderline significant difference among the fabrication techniques in the direct groups (F[2,3]) = 3.231, *p* = 0.050, partial *η* ^2^ = 0.14). Pairwise comparisons indicated that direct casting demonstrated significantly better adaptation than direct milling (*p*  < 0.001), whereas no significant differences were found between casting and SLM or between SLM and milling. In the indirect groups, a significant difference among the fabrication techniques was observed (F[2,39] = 3.697, *p* = 0.034, partial *η* ^2^ = 0.16). Pairwise comparisons revealed superior adaptation of the SLM group compared with the casting group (*p* = 0.036); no significant differences were detected between casting and milling or between milling and SLM (*p*  > 0.05). Comparison of adaptation between the direct and indirect methods across different fabrication techniques demonstrated a significant difference only in the casting group, with the indirect method showing lower adaptation than the direct method (mean difference = −21.75 mg; 95% CI: −27.8 to −15.70; *p*  < 0.001; Cohen’s *d* = 2.08).

**Figure 5:**
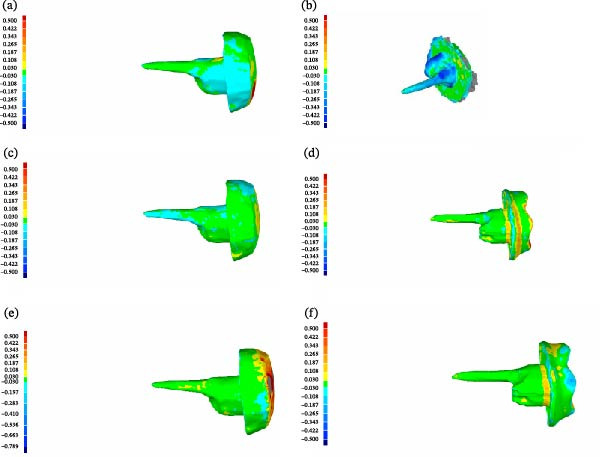
Color map of (a) casting (b) casting indirect (c) milling (d) milling indirect (e) SLM (f) SLM indirect.

For trueness (RMS), significant differences among the fabrication techniques were observed in the direct groups (F[2,39] = 11.755, *p*  < 0.001, partial *η* ^2^ = 0.38). Pairwise comparisons showed that milled post‐and‐core restorations had lower trueness than both cast and SLM post‐and‐core restorations (*p*  < 0.001); whereas no significant difference was found between the casting and SLM techniques (*p*  > 0.05). In the indirect groups, significant differences were also found among the techniques (F[2,39] = 4.841, *p* = 0.013, partial *η* ^2^ = 0.20). Pairwise comparisons indicated that cast post‐and‐core restorations had lower trueness than those fabricated by the SLM and milling techniques (*p* = 0.011); no significant difference was observed between SLM and milling (*p*  > 0.05). A comparison of trueness between the direct and indirect methods across different fabrication techniques demonstrated significant differences in the casting and milling groups. In the casting group, the indirect method showed lower trueness than the direct method (mean difference = −0.04 mm; 95% CI: −0.06 to −0.01; *p* = 0.007; Cohen’s *d* = 0.85); in contrast, in the milling group, the direct method showed lower trueness (mean difference = 0.04 mm; 95% CI: 0.002–0.07; *p* = 0.038; Cohen’s *d* = 0.62). Mean RMS values ranged up to 0.14 mm. Color maps of 3D deviations in each group are demonstrated in Figure 5.

The number and percentage of specimens exceeding the 0.05 and 0.10 mm RMS reference thresholds were also calculated. All specimens (84/84, 100%) exhibited a RMS value greater than 0.05 mm. Regarding the 0.10 mm threshold, 72 out of 84 specimens (85.7%) exceeded this value. At the group level, the proportion of specimens with RMS values greater than 0.10 mm was 71.4% for casting, 92.9% for casting‐indirect, 78.6% for SLM, 85.7% for SLM‐indirect, 100% for milling, and 85.7% for milling‐indirect groups. Interobserver and intraobserver reliability coefficients, calculated using the weighted kappa method, were 0.84 and 0.89, respectively, indicating almost perfect agreement.

## 4. Discussion

This study showed that the fabrication method (direct vs. indirect) and technique (casting, milling, and SLM) affected the apical gap, adaptation, and trueness; therefore, the null hypothesis was partially rejected. For the apical gap, the null hypothesis was not rejected among techniques within either the direct or indirect methods, but it was rejected for the casting group when comparing direct and indirect methods. For adaptation, the null hypothesis was rejected only for casting versus milling in the direct group and for direct versus indirect casting; it was not rejected in other comparisons. For trueness, the null hypothesis was rejected for milling in the direct group and for casting in the indirect group, as well as for direct versus indirect comparisons in casting and milling; it was not rejected for other comparisons, including SLM.

We used a semi‐digital workflow—resin patterns or silicone impressions, followed by laboratory scanning and CAD/CAM fabrication—because it avoids the scan‐post/canal mismatch reported for fully digital approaches. This advantage is likely related to the ability of semi‐digital workflow to more accurately reproduce the internal canal morphology, particularly in narrow or >10‐mm canals. This interpretation is supported by previous studies showing higher accuracy when a semi‐digital workflow is used in such conditions [31, 33, 39, 51].

Leaving 3–5 mm of gutta‐percha is critical to minimize microleakage; any gap between the post tip and this apical seal compromises this barrier [46, 47]. In our radiographic analysis, no technique‐related difference appeared within either fabrication method (direct: *p* = 0.64; indirect: *p* = 0.138). This contrasts with previous fully digital studies of Jafarian et al. [39] and Hendi et al. [31], where cast posts demonstrated better results than milled ones—likely due to inherent limitations of scan‐post workflows, particularly their reduced ability to capture the terminal portion of narrow canals, compared with the semi‐digital resin‐pattern technique used in the present study. Only casting showed a method effect here: direct casting created a smaller gap than indirect casting (*p* = 0.009), probably because each additional impression/investment step in the indirect route compounds dimensional error [52].

Regarding adaptation, tight contact between the post, cement, and canal wall directly enhances micromechanical retention, particularly in the apical third [13, 53]. In our data, direct casting demonstrated better results than direct milling (*p*  < 0.001); whereas, in the indirect groups, SLM showed superior performance than casting (*p* = 0.036). The superior adaptation of cast posts is expected as resin patterns are formed intracanally and can more accurately replicate irregular anatomy; in contrast, milling is constrained by bur diameter and cumulative CAM error [54]. This methodological difference may explain the observed superiority of casting in the direct group. Two earlier studies using fully‐digital or half‐digital workflows reported the same trend [14, 39]; however, Liu et al. [27] evaluating Co–Cr posts, reported that the internal adaptation of SLM was not inferior to that of CAD‐CAM milled or cast posts, indicating no significant differences among the groups, which may be attributed to the use of standardized artificial tooth models rather than extracted natural teeth, as well as the use of two‐dimensional measurements obtained from scanning electron microscopy images that reduce anatomical variability. The higher adaptation of indirect SLM may reflect its layer‐wise fabrication process, which minimizes distortion and provides a finer surface finish compared with investment casting [55].

Laboratory scanning offers a nondestructive, remeasurable way to quantify accuracy in three dimensions [30]. Trueness differed by method and technique in this study. In the direct workflow, milling performed worse than casting and SLM (*p*  < 0.001); whereas, in the indirect workflow, casting performed worse than milling and SLM (*p* = 0.011); milling and SLM performed similarly. This pattern can be explained by the limitations of the milling process itself, particularly the restricted bur access in deep canal regions during direct milling and by wax‐pattern shrinkage plus investment expansion in indirect casting [56]. SLM maintained high trueness in both methods, which may be related to its manufacturing precision; however, this accuracy can be influenced by factors such as machine characteristics and processing parameters [57]. Earlier work is inconsistent: Kanduti et al. [29] reported reduced postcanal space in the apical region (i.e., better fit based on micro‐computed tomography measurements) for cast posts fabricated using a direct casting approach compared with SLM posts produced via a fully digital CAD/CAM workflow; however, Piangsuk et al. [30] found no difference among techniques in an indirect‐only design.

The selection of 0.05 and 0.10 mm as reference thresholds was based on previously reported values in the literature, where marginal discrepancies between 50 and 120 µm have been widely considered clinically acceptable for fixed dental restorations [58–60]. In the present study, all specimens exceeded the stricter 0.05 mm threshold, whereas the majority also exceeded the 0.1 mm threshold, indicating that these values should be interpreted as conservative reference benchmarks rather than absolute failure criteria. Thus, relative differences between methods remain more meaningful for comparisons.

This study had some limitations. First, intraoral temperature, saliva, and functional loading were not replicated because the study was carried out in vitro. Second, the apical gap was measured using two‐dimensional periapical radiographs, which are susceptible to projection errors and buccolingual superimposition. Therefore, this method may not fully reflect the true 3D spatial relationship at the apical region despite standardization and calibration procedures. Third, the weight of the silicone material was measured once under standardized conditions; however, no repeated weighing protocol was applied. Fourth, internal and marginal gaps were not evaluated at all, and adaptation was deduced from the total weight of a light‐body silicone layer—an approach that presumes uniform flow and might miss local voids. This method provides an overall assessment of internal adaptation but does not allow the evaluation of the spatial distribution of the material within the canal. Fifth, the absence of micro‐computed tomography analysis represents an additional limitation, as it would have enabled a more accurate 3D assessment of postcanal adaptation. Finally, the alloys used for the different fabrication techniques were not chemically identical. Differences in alloy composition may influence properties such as shrinkage behavior, thermal expansion, and microstructure, which, in turn, could affect the measured fit parameters. Therefore, some of the observed differences may be partially attributed to material‐related factors rather than the fabrication technique alone. In addition, no metallurgical characterization (e.g., microstructural analysis or hardness testing) was performed, which limits further interpretation of these effects.

To obtain a more complete volumetric picture of post‐and‐core accuracy, future studies should switch to in vivo settings, expand accuracy analysis to include internal and marginal gaps, and use high‐resolution 3D techniques like silicone‐replica/3D superimposition or micro‐computed tomography.

### 4.1. Clinical Implications

These findings provide guidance for clinicians selecting post‐and‐core fabrication methods. For cases where maximal trueness is critical, such as narrow or anatomically complex canals, SLM may demonstrate favorable trueness, but not universal superiority. Conventional casting remains a reliable choice for achieving optimal apical fit, particularly in direct workflows, where it produced smaller apical gaps. Milling can be useful for efficient digital workflows, although slight reductions in trueness may occur, especially in direct workflows for deep canals. Overall, the choice between direct and indirect methods should consider both the desired precision and the clinical availability of digital equipment, allowing treatment to be tailored for optimal fit and feasibility.

## 5. Conclusions

Within the limitations of this in vitro study, the fabrication technique influenced fit parameters under specific circumstances. The apical gap did not differ significantly between techniques within either workflow, although direct casting produced a smaller gap than indirect casting. For adaptation, casting demonstrated better results than milling in the direct workflow; in contrast, SLM showed superior adaptation in the indirect workflow. Regarding trueness, milling was less accurate than casting and SLM in the direct workflow, whereas casting was less accurate than milling and SLM in the indirect workflow. These findings suggest potential trends in post‐and‐core fit, but further in vivo studies are needed to confirm their clinical relevance. Clinically, these results may guide the selection of post‐and‐core fabrication methods based on the canal morphology, required precision, and availability of digital or conventional workflows.

**Nomenclature**
SLM: Selective laser melting
RMS: Root mean square
CAD/CAM: Computer‐aided design/computer‐aided manufacturing
3D: Three‐dimensional
Co–Cr: Cobalt–chromium
STL: Standard‐tessellation‐language
SD: Standard deviation.

## Author Contributions

**Hadi Ranjzad:** conceptualization, methodology, funding acquisition, writing – review and editing. **Jalal Toumaj:** writing – original draft, data curation. **Farzaneh Ostovarrad**: resources, investigation, visualization. **Mehran Falahchai:** methodology, project administration, supervision. **Mahyar Eftekhar:** methodology, data curation, writing.

## Funding

This work was funded and supported by the Dental Sciences Research Center of Guilan University of Medical Sciences (Grant #IR.GUMS.REC.1402.022).

## Ethics Statement

All experimental protocols were approved by the Research Ethics Committees of Guilan University of Medical Sciences. All methods were carried out in accordance with the ethical principles and the national norms and standards for conducting Medical Research in Iran. The ethics code is IR.GUMS.REC.1402.022.

## Consent

All patients provided written informed consent for the use of their extracted teeth in this study.

## Conflicts of Interest

The authors declare no conflicts of interest.

## Data Availability

The data that support the findings of this study are available from the corresponding author upon reasonable request.
